# Disease Progression in MRL/lpr Lupus-Prone Mice Is Reduced by NCS 613, a Specific Cyclic Nucleotide Phosphodiesterase Type 4 (PDE4) Inhibitor

**DOI:** 10.1371/journal.pone.0028899

**Published:** 2012-01-11

**Authors:** Thérèse Keravis, Fanny Monneaux, Issaka Yougbaré, Lucien Gazi, Jean-Jacques Bourguignon, Sylviane Muller, Claire Lugnier

**Affiliations:** 1 CNRS, Biophotonique et Pharmacologie, Faculté de Pharmacie, Université de Strasbourg, Illkirch, France; 2 CNRS, Immunologie et Chimie Thérapeutiques, Institut de Biologie Moléculaire et Cellulaire, Strasbourg, France; 3 CNRS, Laboratoire d'Innovation Thérapeutique, Faculté de Pharmacie, Université de Strasbourg, Illkirch, France; Institut Jacques Monod, France

## Abstract

Systemic lupus erythematosus is a polymorphic and multigenic inflammatory autoimmune disease. Cyclic AMP (cAMP) modulates inflammation and the inhibition of cyclic nucleotide phosphodiesterase type 4 (PDE4), which specifically hydrolyzes cAMP, inhibits TNFα secretion. This study was aimed at investigating the evolution of PDE activity and expression levels during the course of the disease in MRL/lpr lupus-prone mice, and to evaluate in these mice the biological and clinical effects of treatments with pentoxifylline, denbufylline and NCS 613 PDE inhibitors. This study reveals that compared to CBA/J control mice, kidney PDE4 activity of MRL/lpr mice increases with the disease progression. Furthermore, it showed that the most potent and selective PDE4 inhibitor NCS 613 is also the most effective molecule in decreasing proteinuria and increasing survival rate of MRL/lpr mice. NCS 613 is a potent inhibitor, which is more selective for the PDE4C subtype (IC_50_ = 1.4 nM) than the other subtypes (PDE4A, IC_50_ = 44 nM; PDE4B, IC_50_ = 48 nM; and PDE4D, IC_50_ = 14 nM). Interestingly, its affinity for the High Affinity Rolipram Binding Site is relatively low (K_i_ = 148 nM) in comparison to rolipram (K_i_ = 3 nM). Finally, as also observed using MRL/lpr peripheral blood lymphocytes (PBLs), NCS 613 inhibits basal and LPS-induced TNFα secretion from PBLs of lupus patients, suggesting a therapeutic potential of NCS 613 in systemic lupus. This study reveals that PDE4 represent a potential therapeutic target in lupus disease.

## Introduction

Systemic lupus erythematosus (SLE) is a polymorphic and multigenic autoimmune disease that predominantly affects women. The prevalence of lupus in the UK ranges from 40 cases per 100,000 people among northern Europeans to more than 200 cases per 100,000 people in the black population [1]. This inflammatory disease is characterized by the presence of anti-double stranded DNA marker antibodies in the serum of patients and by characteristic lupus nephropathy inducing chronic renal failure. There is no specific treatment for this pathology, which is addressed with symptomatic treatments, such as corticoids and immunosuppressant [2], [3].

Cyclic AMP (cAMP) is a key intracellular second messenger, which is an important modulator of inflammation. Downstream receptor activation, intracellular signalling is regulated by cyclic nucleotide phosphodiesterase families (PDE1 to PDE11) that hydrolyze cAMP and cGMP as a feedback mechanism to return to basal levels, then mediating cAMP-dependent and cGMP-dependent protein kinase activation [4]. Among PDEs, the members of the PDE4 family (PDE4A, 4B, 4C and 4D) specifically hydrolyse cAMP and are mainly present in inflammatory cells [5]. Studies performed with mice deficient in PDE4B demonstrated that this PDE4 family member plays an essential role in TNFα production by peripheral leukocytes and macrophages [6], [7]. Therefore, one could question about the possible participation of PDE4 family members in SLE. This study was designed for investigating the PDE4 expression and activity in MRL/lpr lupus-prone mice and for examining *in vivo* the effects of PDE4 inhibitors on SLE disease progression.

Modifications of cAMP metabolism have been investigated in the kidneys of MRL/lpr lupus-prone mice as the disease progressed by assessing PDE4 activity and expression, PDE4 being the major PDE isozyme regulating cAMP level. The effects of pentoxifylline [8], denbufylline [9], [10] and NCS 613 [11] that differently inhibit PDE4 activity have been studied both *in vivo* in treated animals and *ex vivo*, by testing lipopolysaccharide (LPS)-induced TNFα secretion by peripheral blood lymphocytes (PBLs). NCS 613 effect was also analyzed *ex vivo* on LPS-induced TNFα secretion by PBLs from patients with SLE. This study reveals that PDE4 represents a potential therapeutic target in SLE disease and that NCS 613 treatment delays lupus progression.

## Results

### Evolution with disease progression of cAMP-PDE activities in the kidneys of MRL/lpr mice

**Figure 1:**
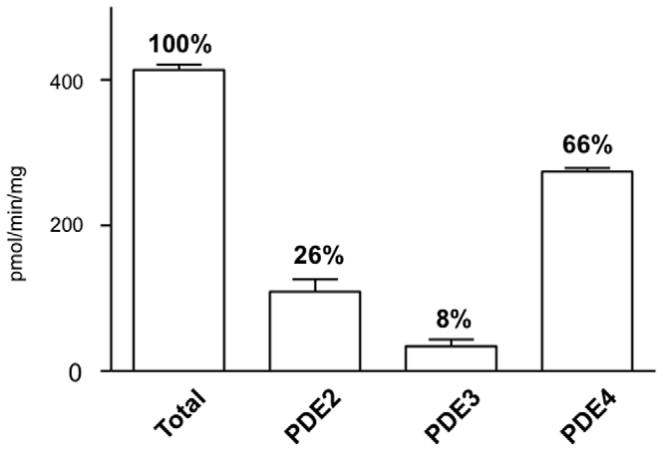
Pattern of cAMP-PDE activities in the kidneys of 8 week-old CBA/J control mice. cAMP-PDE specific activities in total homogenate and contribution of PDE2, PDE3 and PDE4 were assessed as described in the Methods section. Data are expressed as pmol/min/mg of protein and are the mean±s.e.m. of the data obtained from three individual mice.

**Figure 2:**
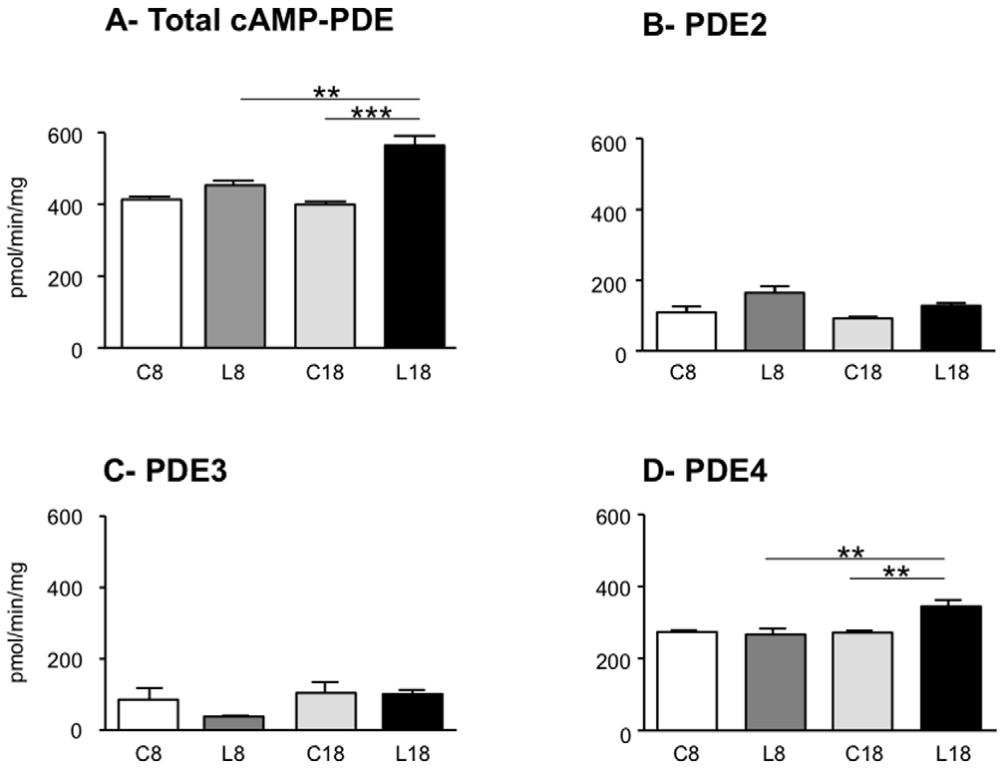
Evolution with the disease of cAMP-PDE activities in the kidneys of MRL/lpr mice. cAMP-PDE specific activities in total homogenate (A) and contribution of PDE2, PDE3 and PDE4 (B–D) were assessed on 8 week-old (C8) and 18 week-old (C18) CBA/J control mice and 8 week-old (L8) and 18 week-old (L18) MRL/lpr mice as described in the Methods section. Data are expressed as pmol/min/mg of protein and are the mean±s.e.m. of the data obtained from three individual mice. **, *P*<0.01; ***, *P*<0.001.

cAMP-PDE activities were assessed in the kidneys of MRL/lpr lupus-prone mice at two time points of the disease progression, namely at 8 weeks, before major changes in survival rate, proteinuria and serum anti-double-stranded DNA antibody levels occur, and at 18 weeks, characterized in this strain by high levels of proteinuria present in 70% of mice and serum anti-DNA antibodies occurring in 90% of animals [12]. Haplotype-matched CBA/J normal mice of the same age were used as control. The pattern of cAMP-PDE activities in kidney extracts of 8 week-old CBA/J mice shows that cAMP hydrolysis is essentially under the control of PDE4 (66%), while PDE2 and PDE3 contribute only for 26% and 8%, respectively (Figure 1). Total cAMP-PDE activity is significantly modified (Figure 2A), as analyzed with a two-way ANOVA test (age phenotype: *P* = 0.0159, *F* = 9.287; disease phenotype: *P* = 0.0002, *F* = 41.20; interaction: *P* = 0.0042, *F* = 15.61). Eight week-old MRL/lpr mice display levels of total cAMP-PDE activity that are similar to those measured in control mice (Figure 2A). In contrast total cAMP-PDE activity levels in the kidneys of 18 week-old MRL/lpr lupus-prone mice are increased by 41% (*P*<0.001) and 24% (*P*<0.01) compared with 18 week-old CBA/J control and with 8 week-old MRL/lpr lupus-prone mice, respectively (Figure 2A). This increase is not due to PDE2 or PDE3 (Figures 2B and 2C) but rather to PDE4 (Figure 2D). PDE4 activity is significantly modified (Figure 2D), as analyzed with a two-way ANOVA test (age phenotype: *P* = 0.0148, *F* = 9.575; disease phenotype: *P* = 0.0302, *F* = 6.91; interaction: *P* = 0.0116, *F* = 10.58). PDE4 activity levels in the kidneys of 18 week-old MRL/lpr lupus-prone mice are increased by 27% (*P*<0.01) and 30% (*P*<0.01) compared with the 18 week-old CBA/J control and with the 8 week-old MRL/lpr lupus-prone mice, respectively (Figure 2D). The changes on PDE4 activity are related to disease progression and not to a simple effect of aging, since no significant alteration was seen between the 8^th^ and 18^th^ week in CBA/J controls.

### Evolution with disease progression of PDE4 protein expression in the kidneys of MRL/lpr mice

**Figure 3:**
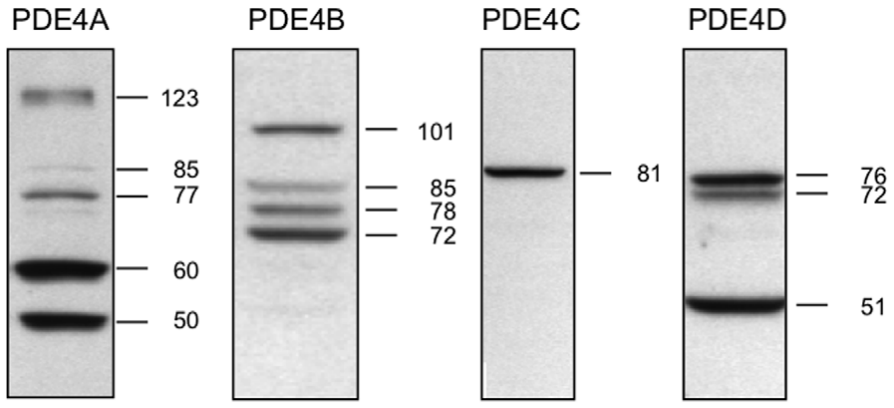
Protein expression pattern of PDE4 in the kidneys of 8 week-old CBA/J control mice. PDE4A, PDE4B, PDE4C, and PDE4D protein expression was assessed on 8 week-old CBA/J control mice as described in the Methods section. The apparent size of protein bands is expressed in kDa.

**Figure 4:**
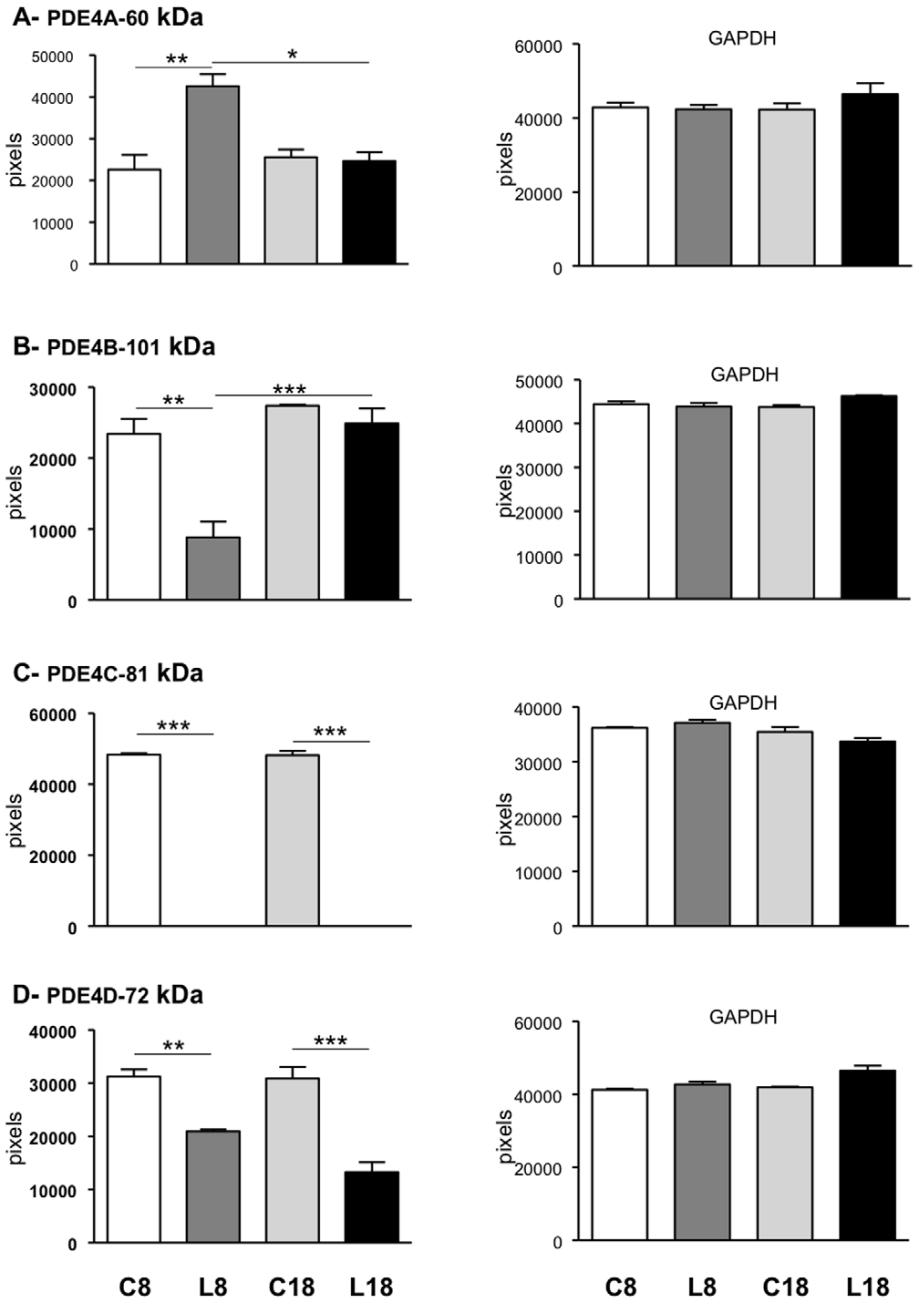
Protein expression pattern of PDE4 in kidneys of MRL/lpr mice of 8 and 18 weeks. PDE4A, PDE4B, PDE4C, PDE4D and GAPDH protein expressions were assessed on 8 week-old (C8) and 18 week-old (C18) CBA/J control mice and 8 week-old (L8) and 18 week-old (L18) MRL/lpr mice. Data are the mean±s.e.m. of three mice.*, *P*<0.05; **, *P*<0.01; ***, *P*<0.001.

To further explore PDE proteins in MRL/lpr lupus-prone mice, kidney extracts were subjected to SDS-PAGE and PDE proteins were analyzed by Western immunoblotting using kidney extracts from CBA/J mice as control. Figure 3 shows that variants of the four PDE4 subfamilies are expressed at different levels in the kidney extracts of 8 week-old CBA/J control mice (quantification in Table 1). Among the variants of the four PDE4 subtypes, several display statistically different expression levels as analyzed with a two-way ANOVA test (Figure 4): (i) PDE4A-60 kDa (age phenotype: *P* = 0.034, *F* = 6.845; disease phenotype: *P* = 0.0216, *F* = 11.09; interaction: *P* = 0.0083, *F* = 13.23); (ii) PDE4B-101 kDa (age phenotype: *P* = 0.0007, *F* = 28.06; disease phenotype: *P* = 0.0019, *F* = 20.50; interaction: *P* = 0.0127, *F* = 10.23); (iii) PDE4C-81 kDa (age phenotype: *P* = 0.9282, *F* = 0.008641; disease phenotype: *P*<0.0001, *F* = 6015; interaction: *P* = 0.9282, *F* = 0.008647); and (iv) PDE4D-72 kDa (age phenotype: *P* = 0.0361, *F* = 6.326; disease phenotype: *P*<0.0001, *F* = 75.30; interaction: *P* = 0.0508, *F* = 5.272). In the kidneys of 8 week-old MRL/lpr mice (L8), the expression of PDE4A-60 kDa protein (87% increase, *P*<0.01; Figure 4A), PDE4B-101 kDa (63% decrease, *P*<0.01; Figure 4B), PDE4C-81 kDa (undetectable, *P*<0.001; Figure 4C), and PDE4D-72 kDa (33% decrease, *P*<0.01; Figure 4D) is affected compared to CBA/J kidneys (C8). In 18 week-old MRL/lpr lupus-prone mice (L18), while PDE4A-60 kDa and PDE4B-101 kDa expression returns to a level similar to CBA/J controls (C18), PDE4C-81 kDa protein remains undetectable (*P*<0.001) and PDE4D-72 kDa expression remains significantly decreased (−58%; *P*<0.001). Again, these effects are not simply linked to aging, since no significant changes are seen between 8 and 18 week-old CBA/J controls (C8 and C18). It is noticeable that PDE4B-101 kDa expression is significantly increased during the course of the lupus disease (*P*<0.001, Fig. 4B). Glyceraldehyde 3-phosphate dehydrogenase (GAPDH) expression is stable in all samples (Figure 4, right panel).

**Table 1:** Distribution of PDE4A, PDE4B, PDE4C and PDE4D variants in the kidneys of 8 week-old CBA/J control mice.

PDE4A		PDE4B		PDE4C		PDE4D	
kDa	%	kDa	%	kDa	%	kDa	%
123	16.6±1.0	101	29.1±1.1	81	99.9±0.7	76	32.8±1.7
85	6.5±0.9	78	12.8±0.5			72	24.5±0.7
77	17.5±0.5	85	20.8±0.3			51	42.6±1.5
60	30.7±0.5	72	37.2±1.6				
50	28.5±1.4						

Data are expressed as the percentage of the sum of all signals expressed in each subfamily and represent the mean±s.e.m. of three independent experiments.

### Effect of PDE4 inhibitors on disease progression of MRL/lpr mice

**Figure 5:**
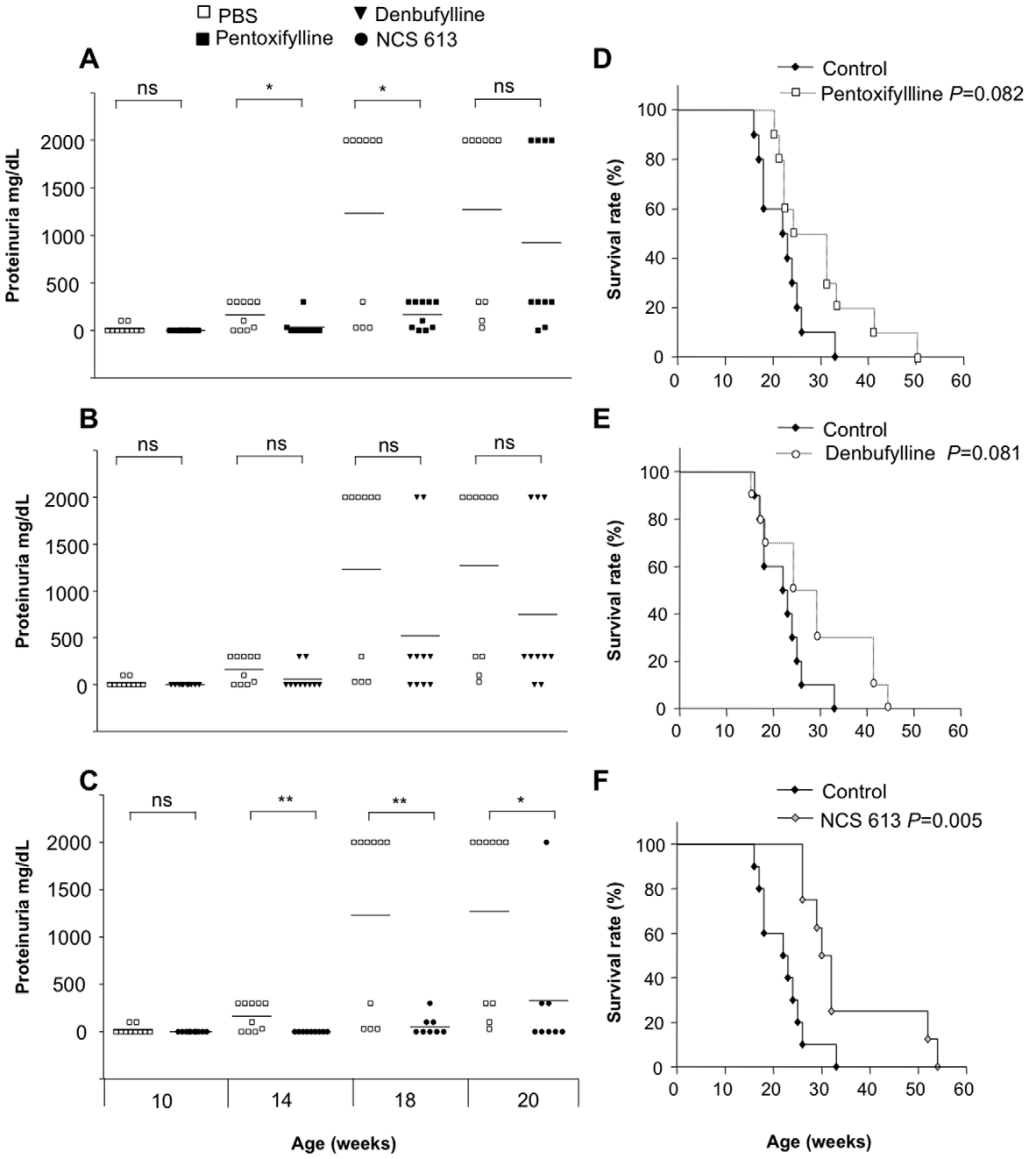
Pentoxifylline, denbufylline and NCS 613 treatment effects on proteinuria and survival rate of MRL/lpr mice. Four groups of MRL/lpr mice were injected via the i.v. route at 5, 7, 9 and 13 weeks with either 100 µL of PBS-10% ethanol (control, n = 10), or 100 µg pentoxifylline in 100 µL PBS-10% ethanol (n = 10), or 100 µg denbufylline in 100 µL PBS-10% ethanol (n = 10), or 30 µg NCS 613 in 100 µL PBS-10% ethanol (n = 8). Proteinuria was measured as described in the Methods section at weeks 10, 14, 18 and 20 are represented for each mouse. Each symbol represents an individual mouse and horizontal lines indicate the median. Closed symbols represent PBS-treated mice and open symbols represent inhibitors-treated mice (pentoxifylline, denbufylline and NCS 613). Because PBS-treated-MRL/lpr mice present a high mortality, dead mice were considered to have a proteinuria >2000 mg/dL (the level measured the week before their death). Survival rate is expressed as the percentage of surviving mice with time expressed in weeks. *, *P*<0.05; **, *P*<0.01, ns; not significant.

MRL/lpr lupus-prone mice have been treated with PDE inhibitors with different selectivity and specificity toward PDE4, namely pentoxifylline characterized by a lack of PDE isoform selectivity, denbufylline displaying a higher selectivity for PDE4, and NCS 613 characterized by a high selectivity for PDE4 and a strong potency (Tables 2 and 3). MRL/lpr mice were injected intravenously (i.v.) with either 30 µg NCS 613, 100 µg denbufylline, or 100 µg pentoxifylline, or the vehicle only. They were monitored regularly for renal disease (checked by the proteinuria level) and survival. Although no significant effect was seen on the level of DNA antibodies measured in the serum of treated mice, animals that received two of the three PDE4 inhibitors (i.e. pentoxifylline and NCS 613) developed a proteinuria level that was significantly lower compared to the control group (Figures 5A,B,C). NCS 613 was the most effective inhibitor in delaying proteinuria. The first mouse that developed proteinuria in the NCS 613 group was 18 week-old (Figure 5C, *P* = 0.005 compared to the control group) while at the same age, around 50% of mice treated with pentoxifylline or denbufylline and 70% of untreated mice had positive proteinuria.

**Table 2:** IC_50_ (µM) values for pentoxifylline, denbufylline and NCS 613 on PDE1-PDE5 isozymes.

Isozyme	PDE1	PDE2	PDE3	PDE4	PDE5	PDE5/PDE4
Substrate Modulator	cGMP CaM	cAMP cGMP	cAMP	cAMP	cGMP	
**Pentoxifylline**	236±20	119±10	84±7	135±11	74±6	0.55
**Denbufyllline**	133±11	208±19	>300	0.76±0.05	5.4±0.5	7.11
**NCS 613**	39±2	24±3	>300	0.042±0.003	4.7±0.1	111.9

IC_50_ values were determined at 1 µM substrate concentration in the presence of the modulator for PDE1 and PDE2 and represent the mean±s.e.m. of three independent experiments. CaM = calmodulin.

**Table 3:** IC_50_ (µM) values for pentoxifylline, denbufylline and NCS 613 on human recombinant PDE4 subtypes.

	PDE4A	PDE4B	PDE4C	PDE4D
Pentoxifylline	99(91–109)	61(54–69)	216(191–244)	45(37–57)
Denbufylline	0.23(0.22–0.25)	0.17(0.15–0.18)	1.21(1.07–1.37)	0.45(0.41–0.50)
NCS 613	0.0436(0.0354–0.0538)	0.0481(0.0443–0.0523)	0.0014(0.0012–0.0016)	0.0144(0.0118–0.0175)

IC_50_ values were determined at 1 µM cAMP substrate concentration and are given with their confident intervals.

More importantly, only the administration of NCS 613 significantly increased survival of MRL/lpr mice (Figure 5F, *P* = 0.005). Mice treated with NCS 613 started dying later, at 26 weeks *vs.* 17 weeks in the control group and 22 and 15 weeks in the pentoxifylline (Figure 5D) and denbufylline (Figure 5E) groups, respectively. At 31 weeks, 50% of NCS 613-treated mice were still alive, when the median survival time for mice treated with PBS, pentoxifylline or denbufylline was 22.5, 27.5 and 26.5 weeks, respectively.

### Effect of PDE4 inhibitors on LPS-induced TNFα secretion by PBLs from MRL/lpr mice

**Figure 6:**
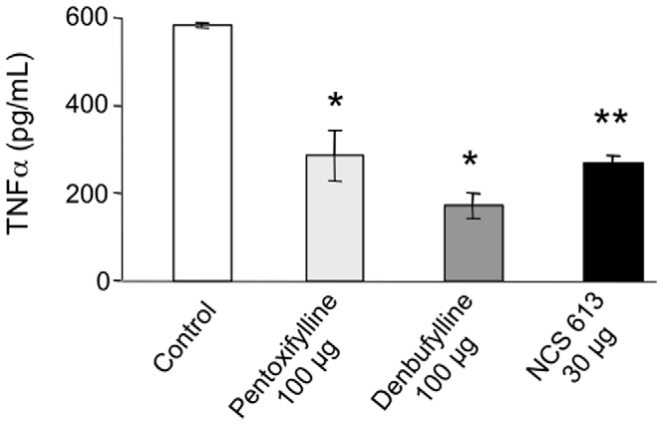
PDE inhibitor treatment effects on *ex vivo* LPS-induced TNFα secretion by PBLs from MRL/lpr mice. Four groups of 7 MRL/lpr mice each were injected i.v. at 5, 7, 9 and 13 weeks with either 100 µL of PBS-10% ethanol (control), or 100 µg pentoxifylline in 100 µL PBS-10% ethanol, or 100 µg denbufylline in 100 µL PBS-10% ethanol, or 30 µg NCS 613 in 100 µL PBS-10% ethanol. Blood samples were collected from 14 week-old treated mice. PBLs pooled from seven mice of each group were purified and cultured in the presence of LPS, and TNFα secretion was determined by ELISA 24 h later. The results are expressed as the mean concentration (pg/mL) ±s.e.m. of duplicate cultures. *, *P*<0.05; **, *P*<0.01.

To assess the impact of different PDE4 inhibitors on LPS activation of cytokine responses, PBLs isolated from treated MRL/lpr mice were incubated with 5 µg/mL LPS and the levels of TNFα were measured in culture supernatants 24 h later. As shown in Figure 6, LPS stimulation induced high levels of TNFα secretion by PBLs from MRL/lpr mice that received vehicle only. Interestingly, LPS-induced TNFα production was significantly decreased in the cultures of PBLs from MRL/lpr mice treated with PDE4 inhibitors (*P*<0.05). The calculated inhibition rate of TNFα secretion was in the same range in all groups (51% for pentoxifylline, 70% for denbufylline and 54% for NCS 613).

### Effect of NCS 613 on LPS-induced TNFα secretion by PBLs from SLE patients

**Figure 7:**
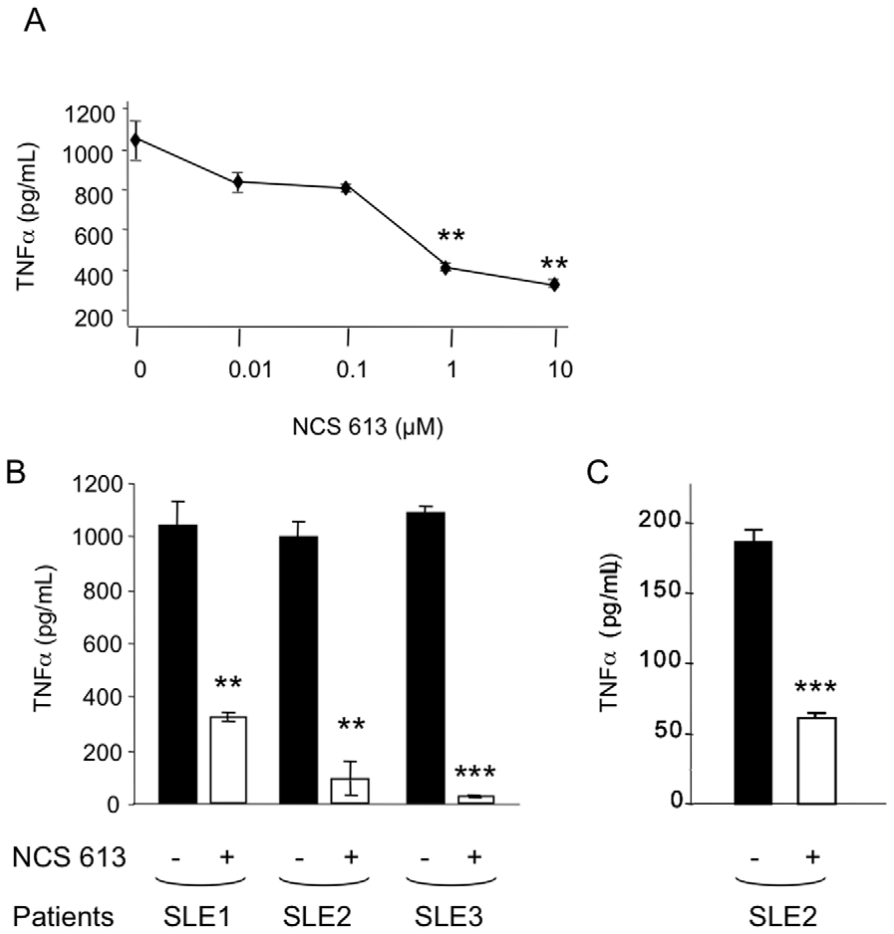
Effect of NCS 613 on LPS-induced and basal TNFαsecretion by PBLs from SLE patients. PBLs from three patients with SLE (SLE1, SLE2 and SLE3) were purified and cultured as described in Materials and Methods. PBLs from SLE1 patient were incubated for 45 min with increasing doses of NCS 613 and stimulated with 5 µg/mL of LPS (**A**). PBLs were incubated for 45 min with (□) or without (▪) 10 µM of NCS 613 and stimulated (**B**; SLE1, SLE2 and SLE3) or not (**C**; SLE2) with 5 µg/mL of LPS. TNFα secretion was determined by ELISA. The results are expressed as the mean concentration (pg/mL) ±s.e.m. of duplicate cultures. There was no basal TNFα secretion in the culture of PBMCs from SLE1 and SLE3 patients. *, *P*<0.05; **, *P*<0.01; ***, *P*<0.001.

We then extended our studies to assess the effect of NCS 613 on LPS-induced TNFα secretion by PBLs from lupus patients. In general, the latter have blood disorders and particularly leucopenia and lymphopenia. This feature precludes performing complete dose-response measurements *ex vivo*. Therefore, in a preliminary experiment we selected a sample for which we could benefit from a sufficient number of cells to determine the amount of NCS 613 required in this assay. As expected, after 24 h incubation, LPS induced a marked production of TNFα by PBLs from this patient (patient SLE1; 1035±91 pg/ml vs 15±6 pg/ml without LPS), and this production was significantly suppressed by 1 and 10 µM of NCS 613 (*P* = 0.01 and 0.008 respectively; Figure 7A). No effect was seen at a lower NCS 613 concentration (0.1 µM). The following tests were thus performed using 10 µM of NCS 613 (Figure 7B). NCS 613 significantly inhibited 70 to 98% of LPS-induced TNFα production from PBLs originated from SLE patients (SLE1, *P* = 0.008; SLE2, *P* = 0.005; SLE3, *P* = 0.0002). NCS 613 also decreased the basal TNFα secretion level by 67% in the case of cells from patient 2 (*P* = 0.0004; Figure 7C).

### Determination of NCS 613 K_i_ value on ^3^H-rolipram binding site

Rolipram was the first specific PDE4 inhibitor chemically synthesized. Since most of the rolipram analogs and rolipram itself induce emesis, and since this effect was found to be related to their interaction with the so-called High Affinity Rolipram Binding Site (HARBS; [13]–[16]), we investigated the capacity of NCS 613 to displace ^3^H-rolipram binding on HARBS. While the K_i_ value of rolipram toward ^3^H-rolipram binding on rat brain membrane was 3 nM (2.4 to 3.9 nM), the K_i_ value of NCS 613 on HARBS was 148 nM (113 to 196 nM) (Figure S1).

### Effects of pentoxifylline, denbufylline and NCS 613 on PDE1-PDE5 isozymes

Denbufylline is a structural analogue of pentoxifylline with some substitutions on the xanthine ring while NCS 613 is an adenine analogue (Table S1). The three molecules have a very low pKa (<1.7). At physiological pH, there is only protonation state and all three are neutral. All three molecules were shown to have moderate lipophilicity (log D_7.4_ values 0.27–3.78;Table S1) indicating they have a good balance between solubility and permeability. Table 2 compares the potency and selectivity of these compounds. Pentoxifylline is a poor PDE inhibitor acting in 10^−5^–10^−4^ molar range concentration, displaying a higher selectivity for PDE5 compared to PDE4 (PDE5≥PDE3≥PDE2 = PDE4>PDE1) with an IC_50_ value of 74 µM for PDE5 and a PDE5/PDE4 ratio value of 0.55 indicating a better selectivity for PDE5 than for PDE4. Denbufylline is a better PDE inhibitor than pentoxifylline, displaying a higher selectivity for PDE4 (PDE4>PDE5>PDE1>PDE2>>PDE3) with an IC_50_ value of 0.76 µM for PDE4 and a PDE5/PDE4 ratio value of 7.11. Compared to pentoxifylline, the substitutions on the xanthine ring of denbufylline are able to reverse the PDE isozyme selectivity (PDE5 for pentoxifylline and PDE4 for denbufylline) and to produce a much higher potency toward PDE4 (IC_50_ values of 135 and 0.76 µM for pentoxifylline and denbufylline, respectively). NCS 613 is the most potent (IC_50_ value of 0.042 µM; [11]) and the most selective (112-fold relatively to PDE5 and 930-fold relatively to PDE1) PDE4 inhibitor.

### Effects of pentoxifylline, denbufylline, NCS 613 on human recombinant PDE4 subtypes

The PDE4 family comprises PDE4A, PDE4B, PDE4C and PDE4D proteins. As the PDE4D-knocked-out mice developed emesis more easily compared to the wild-type mice, PDE4D inhibition seemed to favor emesis [17]. Therefore, we investigated the subtype PDE4 selectivity of pentoxifylline, denbufylline and NCS 613 (Table 3, Figure S2). As expected, pentoxifylline inhibited PDE4 subtypes in high concentration ranges (10^−5^–10^−4^ M) with IC_50_ values order: PDE4D≈PDE4B<PDE4A<PDE4C. Denbufylline inhibited more potently PDE4 subtypes in the 10^−7^–10^−6^ M concentration range with IC_50_ values order: PDE4B≈PDE4A<PDE4D<PDE4C. Interestingly, NCS 613 selectively inhibited PDE4C with an IC_50_ value of 1.4 nM, in comparison with PDE4D-IC_50_ (14.4 nM), PDE4A-IC_50_ (44 nM) and PDE4B-IC_50_ (48 nM) (Table 3, Figure S2). Furthermore, it should be noticed that both pentoxifylline and denbufylline, which are xanthine analogues, poorly inhibited PDE4C subtype in comparison to other PDE4 subtypes.

## Discussion

The experiments described here in MRL/lpr lupus-prone mice aimed at defining the therapeutic potential of PDE4 inhibitors in this autoimmune disease, which is characterized by nephropathy and inflammatory processes. It is well established that PDE4 inhibitors are anti-inflammatory drugs [15] and that PDE4 inhibitors are beneficial in nephropathy [18], suggesting that PDE4 inhibitors might be helpful for prevention and treatment of SLE.

This study reveals that both the appearance of proteinuria and the survival time of NCS 613-treated MRL/lpr mice are significantly delayed. For obvious reasons of timing, the effect of NCS-613 was not evaluated in strains of mice of different haplotypes such as MRL^+/+^, B6/lpr or (NZBxNZW)F1 mice that develop a spontaneous disease that is significantly slower compared to MRL/lpr mice. It is worth noting also that compared to these mouse models, MRL/lpr mice develop a very strong lupus disease and therefore that any improvement of their clinical and biological signs is highly significant. Our recent data have shown that MRL/lpr mice represent excellent model for translational studies from mice to human [12], [19].

First, we characterized PDE isozyme profile in renal tissue of normal mice and investigated changes in activity and expression in MRL/lpr mice, a mouse model developing a severe lupus disease. In the kidneys of CBA/J mice, PDE4 was found to be the main contributor of cAMP hydrolytic activity, whereas PDE2 and PDE3, which both hydrolyze cAMP and cGMP, contribute to a lower extend. No change with age in total cAMP-PDE activity was seen in 8 and 18 week-old control mice. However, a significant increase (+41%, *P*<0.001) in total cAMP-PDE activity in 18 week-old MRL/lpr mice was seen that was associated to a significant increase in PDE4 activity (+30%, *P*<0.01), indicating that lupus disease specifically alters PDE4 in kidney. Western immunoblotting analysis of PDE4 subtypes revealed the presence of multiple variants in PDE4A, PDE4B and PDE4D families and one variant for PDE4C contributing in total PDE4 activity, attesting the preponderant and complex participation of PDE4 s in renal functions in which multiple micro-compartmentation of these variants might regulate specific processes [4], [20]. Changes in PDE4 variant expression were observed in MRL/lpr mice. Interestingly, the expression of PDE4B-101-kDa increases with the disease and might partially contribute to the increase in PDE4 activity. Indeed, PDE4 activity was raised in 18 week-old mice and the expression of only one PDE4 variant in each subtype family was decreased. It should be noticed that PDE4A-60-kDa represent only 30% of detected PDE4A expression, PDE4B-101-kDa represent only 29% of detected PDE4B expression and PDE4D-72-kDa represent only 24% of detected PDE4D expression. A decrease of the expression of these variants might contribute only weakly to total PDE4 activity. Interestingly, PDE4C-81-kDa expression was undetectable in the renal tissue of MRL/lpr mice even in young mice. At this stage, it is difficult to raise any definitive conclusion on the possible consequences of this drop of PDE4C-81 kDa protein detection. An hypothesis could be that PDE4C is altered in a way that would change its antigenic properties, explaining the lack of recognition by antibody. The contribution of PDE4C into PDE4 activity is considered as minor although it has never been really addressed. It should be noticed that PDE expression does not necessarily reflect PDE protein activity, since some post-translational modifications occur in PDEs, notably PKA-dependent phosphorylation, which might increase PDE4 activity [4], [20], [21]. This question is difficult to solve as no potent and selective PDE4 subtype inhibitor allowing assessing PDE4 subtype contribution in PDE4 activity is available [22].

Our data thus show for the first time that in the kidneys of 18 week-old MRL/lpr mice there is a global increase of PDE4 activity. This prompted us to study the therapeutic potential of PDE4 inhibitors in this mouse model of lupus. Three kinds of compound inhibiting PDE4 were chosen: an anti-inflammatory compound, pentoxifylline, delivered on the pharmaceutical market as Torental® that has previously been studied in MRL/lpr mice [23] and mice with 16/6 Id-induced experimental lupus [24]; denbufylline, a xanthine analogue of pentoxifylline known as PDE4 inhibitor [9], [10] with anti-TNFα property [25], [26] and NCS 613, an adenine analogue, designed and synthesized in our laboratory. NCS 613 is a potent and selective inhibitor of PDE4 [11], which induces *in vivo* and *ex vivo* anti-inflammatory effects [27], [28]. We recently showed in human lung tissues that NCS 613 significantly decreased PDE4 activity and reduced I-ΚBα degradation with a lower expression level of PDE4B and PDE4C [29]. NCS 613 inhibits LPS-induced TNFα secretion by human PBLs with an IC_50_ value of 18 nM [27]. Interestingly, our present data on PDE4 subtypes show that NCS 613, an adenine analogue, is selective for PDE4C and point out that both pentoxifylline and denbufylline, which are xanthine analogues, act differently from NCS 613 on PDE4C since they act very poorly on PDE4C subtypes in comparison to the other PDE4 subtypes.

This study reveals that both the appearance of proteinuria and the survival time of NCS 613-treated mice are significantly delayed. Therefore, in agreement with our previous data obtained in rat kidney with rolipram [18], protective effect of NCS 613 may be linked to its high selectivity and potency for PDE4. We cannot rule out however that *in vivo*, NCS 613 interacts with several PDE4 subtypes (PDE4C>PDE4D>PDE4A = PDE4D) and that NCS 613 restores the PDE4C protein expression. NCS 613 has a direct effect on cAMP degradation but it might also induce long-term changes in PDE4 regulation or expression, as previously shown with rolipram [30].

TNFα secretion that participates in inflammatory processes characterizes lupus progression. Interestingly, denbufylline, pentoxifylline and NCS 613 treatments of MRL/lpr mice significantly decrease LPS-induced TNFαsecretion measured *ex vivo*. Denbufylline, pentoxifylline and NCS 613 display the same effectiveness, keeping in mind that due to its limit of solubility, NCS 613 was given at a three-fold lower dose than the other two drugs. NCS 613 (10 µM) also significantly decreases LPS-induced TNFα secreted by PBLs from unselected patients with SLE. NCS 613, decreases also by 67% the level of basal TNFα secretion, suggesting that NCS 613 might have some potential in the treatment of SLE.

PDE4 inhibitors elicit a number of side-effects, notably emesis, thus limiting their therapeutic potential. Human area postrema and other nuclei related to the emetic reflex express PDE4B and PDE4D, and thus cAMP-signalling modification in the area postrema could mediate the emetic effect of PDE4 inhibitors in human brain stem [31]. The side-effects concern might be alleviated for example by the design of small-molecule allosteric modulators of PDE4D that do not completely inhibit enzymatic activity [32], by the finding of compounds with high potency such as roflumilast [33] and GEBR-7b [34]. An important finding in our work was to demonstrate that, in contrast to rolipram, NCS 613 which efficiently inhibits PDE4 hydrolytic activity, displays a relatively weak ability to bind HARBS. Our result strengthens our previous data showing that NCS 613 does not induce acid gastric secretion [27]. Also, in opposite to rolipram and other PDE4 inhibitors [15], it suggests that NCS 613 might induce very low emetic effects in agreement with the proposal of Souness and Rao [35] and the correlation observed between dose required to induce emesis and that to occupy HARBS [36].

Since we showed that changes in PDE4 s occur in murine lupus and that NCS 613 significantly delayed lupus development, we questioned whether NCS 613 could affect specific PDE4 subtypes. Our study on recombinant PDE4 s reveals that NCS 613 potently and selectively inhibits PDE4C subtype (IC_50_ value of 1.4 nM). Most interestingly NCS 613 targets the PDE4 variant that was found particularly altered in lupus disease, opening a possible avenue for investigation. This low-molecular weight chemical compound (<500 Da) presents also the advantage to be easily administrated and was shown to be active *in vivo per os* on inflammation [27].

In conclusion, this study shows for the first time, to our knowledge, that PDE4 activity is increased in lupus conditions and that among the different PDE4 inhibitors tested, NCS 613, a highly selective PDE4C inhibitor, significantly prevents disease progression by decreasing proteinuria, lowering *ex vivo* TNFα secretion by PBLs and increasing animal survival rate. NCS 613 also inhibits basal and LPS-induced TNFα secretion by PBLs from SLE patients. Although future investigation is warranted to clarify the exact molecular actions of NCS 613, the present data indicate that NCS 613 might have a potential for treating lupus patients.

## Materials and Methods

### Materials

cAMP and cGMP were from Sigma (St. Louis, MO, USA). [8-^3^H] cAMP (25–40 Ci/mmol; 1 mCi/mL) and [8-^3^H] cGMP (5–15 Ci/mmol; 1 mCi/mL) were purchased from Perkin Elmer (Courtaboeuf, France) and purified by thin layer chromatography on silica gel, using isopropanol/NH_4_OH/H_2_O (70/15/15) as a solvent [37]. Tritiated rolipram (23 Ci/mmol; 5 mCi/mL) was a gift from Celltech Therapeutics Society (Slough, UK). Calmodulin was purified from bovine brain as described [38]. Denbufylline and pentoxifylline were generous gifts from Beecham-Wulfing (Gronau, Germany) and Hoechst (Puteaux, France), respectively. NCS 613, cilostamide and rolipram were synthesized as described previously [11], [39], [40]. Anti-PDE4A (AC55) and anti-PDE4B (K118) antibodies [41] were a gift of Dr Marco Conti (Stanford University, USA). Anti-PDE4C (PD4-301AP) and anti-PDE4D (PD4-401AP) antibodies were from FabGennix (Frisco, TX, USA) and anti-GAPDH antibody was from Chemicon (Billerica, MA, USA). Horseradish peroxidase-conjugates were from Promega (Charbonnières-les-Bains, France). ECL kit was from GE Healthcare (Orsay, France).

### Animals

Female CBA/J (H-2^k^) and MR/lpr (H-2^k^) mice were purchased from Harlan (Gannat, France). The animal experimentation was conducted according to the “Principles of Laboratory Animal Care” and with the approval of the Regional Ethics Committee of Strasbourg (CREMEAS, project n°AL/05/08/03/07)

### SLE Patients

Blood samples were obtained from three unselected patients with SLE. The latter fulfilled the American College of Rheumatology criteria for the disease. All samples were obtained from volunteers attending the Rheumatology Clinic of Strasbourg University Hospitals and were collected during routine clinical (diagnostic/prognostic/therapeutic) procedures. Informed verbal consent was obtained from each individual in agreement with the Helsinki declaration and French legislation (article L1221-8-1), under which no approval by an ethical committee was required in this case. Patients were treated by low doses (median dose 10 mg; range 0–20 mg) of methotrexate, hydroxychloroquine and/or non-steroid anti-inflammatory drugs.

### Treatment of lupus-prone mice with PDE inhibitors

A preliminary experiment was performed to investigate the effects of the compounds as well as the influence of the solvent PBS-10% ethanol administrations by the i.v. route. Mice were monitored regularly during 2 weeks following administration and sacrificed for organ observation. No deleterious effects were observed in these conditions of administration.

Four groups of five week-old female MRL/lpr mice were injected via the i.v. route at 5, 7, 9 and 13 weeks with either 100 µL of PBS-10% (v/v) ethanol (control, n = 10), or 100 µg pentoxifylline in 100 µL PBS-10% ethanol (n = 10), or 100 µg denbufylline in 100 µL PBS-10% ethanol (n = 10), or 30 µg NCS 613 in 100 µL PBS-10% ethanol (n = 8); in these conditions, NCS 613 was not soluble at 100 µg/100 µL.

All mice were monitored regularly for different clinical and biological parameters over 26 weeks. Measurements of outcome included the evaluation of survival and proteinuria measured on a fresh urine sample. Protein levels were determined using colored strips (Albutix; Bayer Diagnostics, Basingstoke, UK) and scored at values corresponding to 30, 100, 300 and >2000 mg/dL.

### Measurement of LPS-induced TNF-α secretion by PBLs from MRL/lpr mice

Blood samples were pooled from groups of seven 14 week-old treated mice. PBLs were purified by density separation (Lympholyte-M, d = 1.0875; Cedarlane, Hornby, Canada), washed three times, and resuspended at 5×10^6^ cells/ml in L-alanyl-L-glutamine-enriched RPMI 1640 medium (Cambrex, Verviers, Belgium) containing 10% (v/v) fetal calf serum (Dutscher, Brumath, France), HEPES, gentamycin and β-mercaptoethanol. They were cultured in duplicate using 5×10^5^ cells/well in the presence of 5 µg/mL LPS from E. coli. Culture supernatants were collected after 24 h and stored frozen at −20°C. TNFα levels were determined using a double-sandwich ELISA (PharMingen, San Diego, CA; detection limit 30 pg/mL) according to the manufacturer's instructions.

### Measurement of TNFα secreted by PBLs from SLE patients

PBLs were isolated by centrifugation on Ficoll-Histopaque (Sigma-Aldrich) and cultured as described above using 5×10^5^ cells/well. PBLs were incubated for 45 min with or without 10 µM NCS 613, then stimulated or not with 5 µg/mL LPS. Culture supernatants were collected 24 h later and stored frozen at −20°C before TNFα level determination, as described above.

### Preparation of kidney extracts

CBA/J and MRL/lpr mice were sacrificed by cervical dislocation at 8 and 18 weeks. Kidneys were isolated, immediately frozen in liquid N_2_ and stored at −80°C. Frozen kidneys were powdered-ground in liquid nitrogen using a mortar/pestle set-up. The resulting tissue-powders were homogenized with a glass-glass potter for 3×30 sec at 4°C in the following buffer: 20 mM Tris, pH 7.5, 5 mM EGTA, 150 mM NaCl, 20 mM Na β-glycerophosphate, 1 µM H-89, 10 mM NaF, 1 mM NaVO_3_, 1% (v/v) Triton X-100, 0.1% (v/v) Tween 20, 166 µM Pefabloc, 133 µM aprotinin, 8.3 µM bestatin, 2.5 µM E64, 3.3 µM leupeptin and 1.6 µM pepstatin A. The homogenates were centrifuged at 14,000 *g* for 10 min at 4°C and the supernatants were stored as aliquots at −80°C until used. Protein concentration was determined following Lowry et al. [42] using a compatible detergent assay.

### Measurement of PDE activity in kidney extracts

PDE activity was determined with a radioenzymatic assay as described previously [37]. Total cAMP-PDE activity was assessed at 1 µM cAMP and the contribution of PDE isozymes was determined by using selective inhibitors, 1 µM cilostamide for PDE3 and 10 µM rolipram for PDE4, the residual cAMP-PDE activity representing essentially PDE2. Specific activities were expressed as pmol.min^−1^.mg^−1^ protein.

### Analysis of protein expression pattern of PDE4

Proteins (30 µg) from kidney extracts were subjected to Western immunoblotting as described previously [30]. Briefly, protein samples were denatured and solubilized for 5 min at 95°C in Laemmli buffer, subjected to electrophoresis on SDS-8% polyacrylamide gel and electrotransferred onto polyvinylidene fluoride membranes. Immunodetection was carried out with anti-PDE4A (AC55; 1/2,000), anti-PDE4B (K118; 1/2,000), anti-PDE4C (PD4-301AP; 1/2,500), anti-PDE4D (PD4-401AP; 1/2,500), and anti-GAPDH (1/60,000) antibodies. Immobilized antigens were detected by chemiluminescence using horseradish peroxidase-labelled secondary antibodies (1/60,000), an ECL kit and autoradiography films. Autoradiography signals were captured on a GeneGenius Bio Imaging System (Syngene/Ozyme, Saint Quentin Yvelines, France) using the GeneSnap software and analyzed using the GeneTools software (Syngene/Ozyme). Data are expressed in pixel units.

### Measurement of IC_50_ on purified PDE isoforms

PDE1, PDE3, PDE4 and PDE5 were isolated by anion exchange chromatography from bovine aortic smooth muscle cytosolic fraction [43]. PDE2 was isolated from human platelets following the method described in [44]. Purified PDEs were stored as small aliquots at −80°C until use. PDE activity was determined at a substrate concentration of 1 µM cAMP or cGMP in the presence of 10,000 cpm [^3^H]-cAMP or -cGMP as tracers. PDE1 activity was assessed at 1 µM cGMP in calmodulin-activated state (18 nM calmodulin with 10 µM CaCl_2_). PDE2 activity was assessed at 1 µM cAMP in activated state (+5 µM cGMP), and PDE3 and PDE4 activities were assessed at 1 µM cAMP in the presence of 1 mM EGTA. To prevent reciprocal cross-contamination between PDE3 and PDE4, the assays were carried out in presence of 50 µM rolipram for PDE3 or 50 µM cGMP for PDE4. PDE5 activity was measured at 1 µM cGMP in the presence of 1 mM of EGTA. Denbufylline, pentoxifylline and NCS 613 were dissolved in dimethyl sulfoxide (DMSO). The final concentration of DMSO did not exceed 1% (v/v) for PDE activity assessment. This concentration of DMSO had no effect when tested in control preparation. Denbufylline, pentoxifylline and NCS 613 IC_50_ values were also determined on human recombinant PDE4A, PDE4B, PDE4C and PDE4D kindly given by Ted J. Torphy (SmithKline Beecham Pharmaceuticals, King of Prussia, PA, USA).

The concentration of compounds that produced 50% inhibition of substrate hydrolysis (IC_50_) was calculated by non-linear regression analysis (GraphPad Prism, San Diego, CA) of concentration-response curves including at least 6 different concentrations of inhibitors.

### ^3^H-rolipram binding assay

Binding assays were performed on rat brain membranes with a modification of the method of Schneider et al. [45]. The assay was done in 50 mM N-Tris hydroxymethyl methyl-2-aminoethane sulfonic acid/NaOH buffer, pH 7.6, containing 10 mM MgCl_2_, 0.1 M NaCl and 100 µM 5′AMP. The final volume of reaction mixture was 400 µL containing 4 nM ^3^H-rolipram. For saturation binding, the concentration of ^3^H-rolipram ranged from 0.3 to 12 nM. Non-specific binding was determined in the presence of 1 µM unlabeled rolipram in the incubation medium. Binding assays were started by the addition of 100 µL of rat brain membrane (0.9 mg protein/mL) to the reaction mixture and conducted at 23°C for 1 h under agitation and stopped by the addition of 3 mL of ice-cold reaction buffer and rapid vacuum filtration through Whatman GF/B filters that have been soaked in 0.3% (v/v) polyethylenimine. Filters were washed twice (3 ml of ice-cold reaction buffer without 5′AMP), dried and counted by liquid scintillation. Non-specific binding was consistently lower than 20% of the specific binding. ^3^H-rolipram was bound with high affinity (Kd = 1.4.±0.2 nM) with an Hill number = 1.04±0.05. K_i_ and confidence interval values of binding studies were determined by using one site fit K_i_ GraphPad Prism5 analysis.

### Evaluation of pKa and logD_7.4_ values of denbufylline, pentoxifylline and NCS 613

pKa and log D_7.4_ values were determined by classical procedures by the “plateforme de Chimie Biologique Intégrative de Strasbourg-Technologies du médicament” (PCBiS; http://www.pcbis.fr/).

### Statistical analysis

PDE activity and expression data are expressed as mean±s.e.m. from three independent experiments and analyzed with the two-way ANOVA test with the Bonferroni post-test. TNF-α secretion data were analyzed with the Student's *t*-test for unpaired data. Survival data were analyzed by the Kaplan-Meier product-limit method, with analysis by log rank test. Significance was defined as *P≤*0.05.

## Supporting Information

Table S1 **Chemical structures and some characteristics of pentoxifylline, denbufylline and NCS 613.** The pKa and logD_7.4_ values were determined as described in Materials and Methods.
(TIFF)

Figure S1 **Effects of rolipram (•) and NCS 613 (▪) on ^3^H-rolipram binding were determined as indicated in** **Materials and Methods** **.**
(TIFF)

Figure S2 **Effects of pentoxifylline (A), denbufylline (B) and NCS 613 (C) on human recombinant PDE4 subtypes: PDE4A (•), PDE4B (▪), PDE4C (▾) and PDE4D (♦). IC_50_ values were determined as indicated in** **Materials and Methods** **.**
(TIFF)
