# Supplementary figures and images for: Disease Progression in MRL/lpr Lupus-Prone Mice Is Reduced by NCS 613, a Specific Cyclic Nucleotide Phosphodiesterase Type 4 (PDE4) Inhibitor
Source: PLoS One. 2012 Jan 11;7(1):e28899. doi: 10.1371/journal.pone.0028899 (PMC3256138; doi:10.1371/journal.pone.0028899)

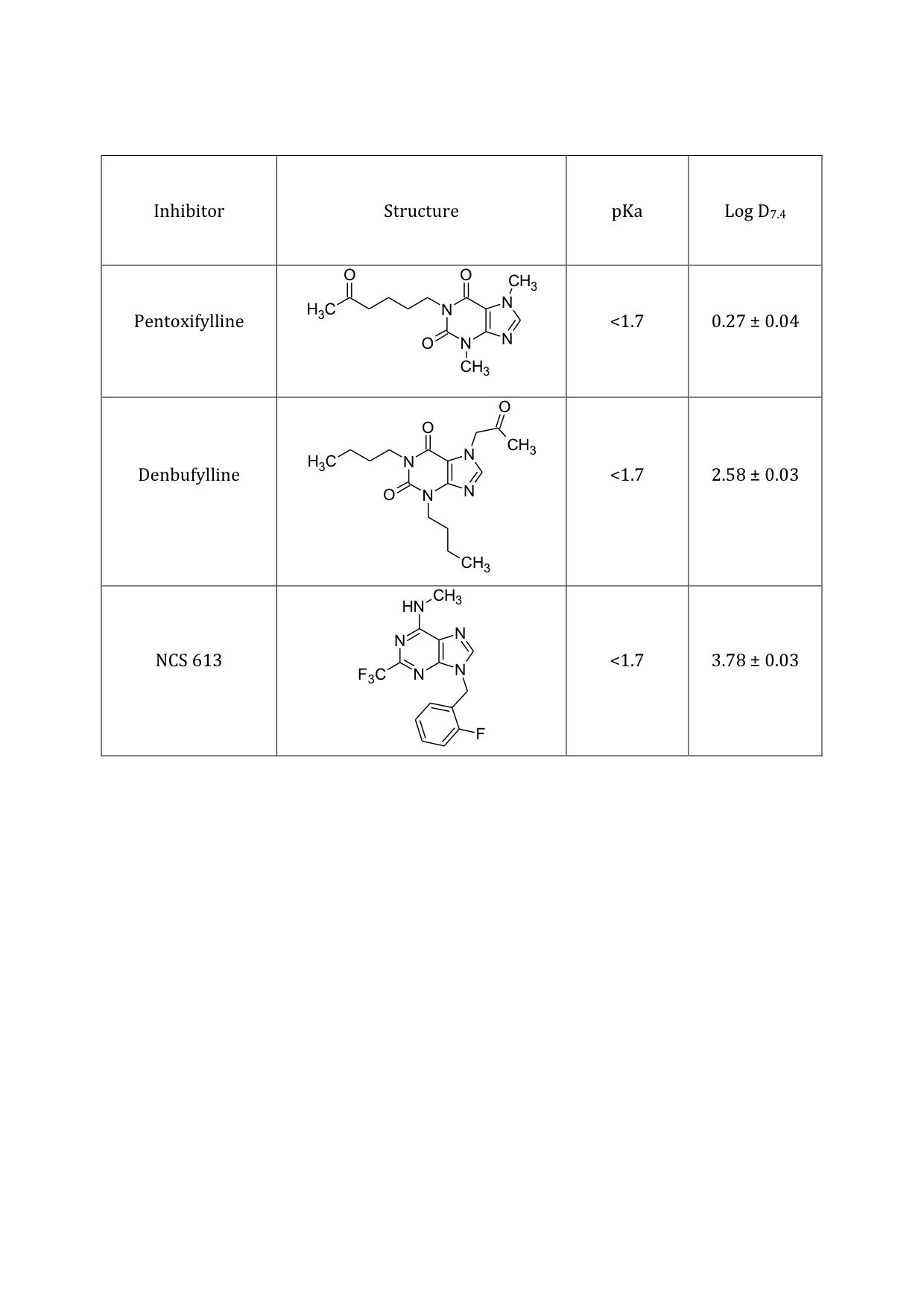

Supplement: Table S1 — Chemical structures and some characteristics of pentoxifylline, denbufylline and NCS 613. The pKa and logD7.4 values were determined as described in Materials and Methods. (TIFF) [file pone.0028899.s001.tif]

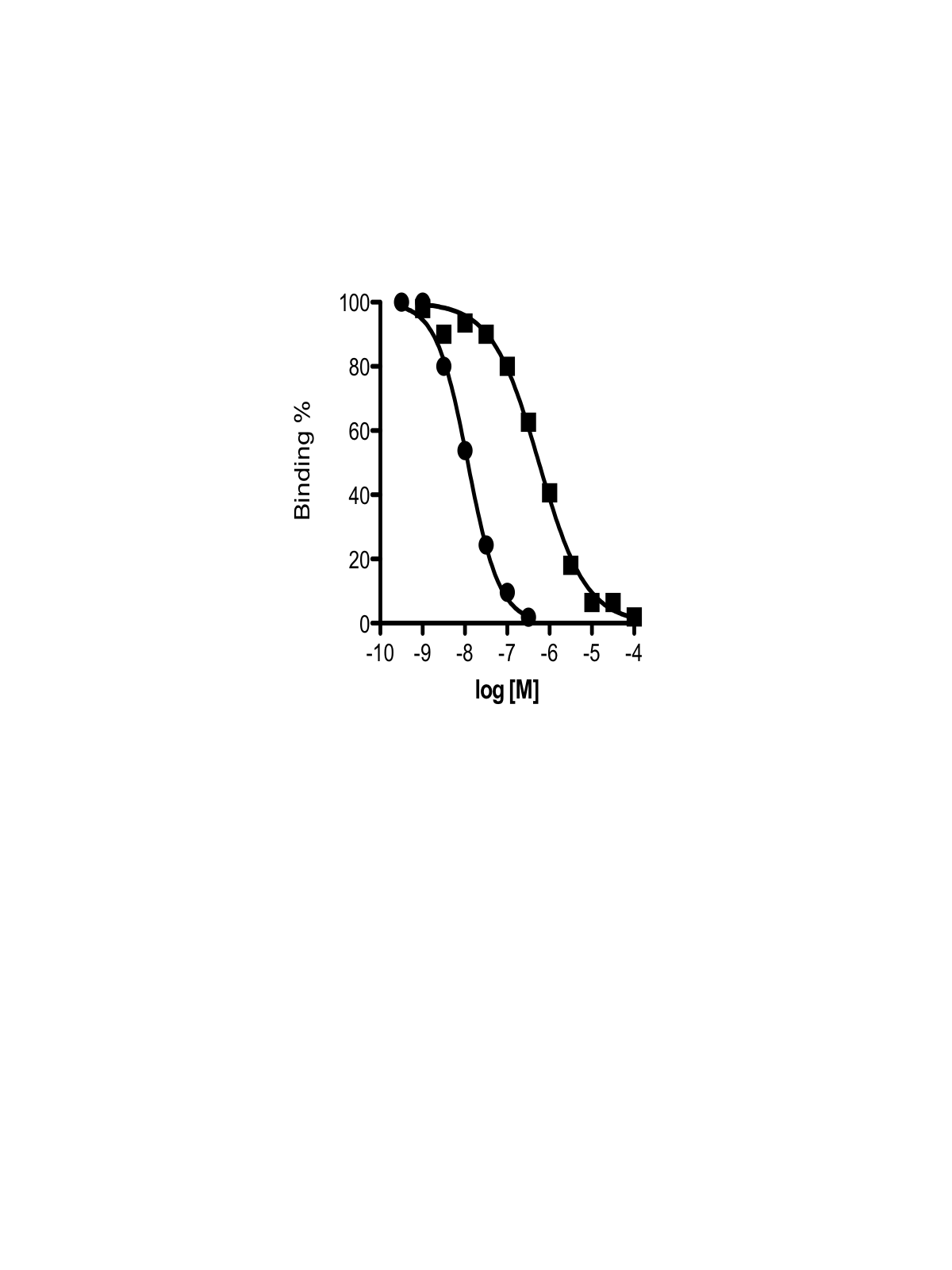

Supplement: Figure S1 — Effects of rolipram (•) and NCS 613 (▪) on 3H-rolipram binding were determined as indicated in Materials and Methods . (TIFF) [file pone.0028899.s002.tif]

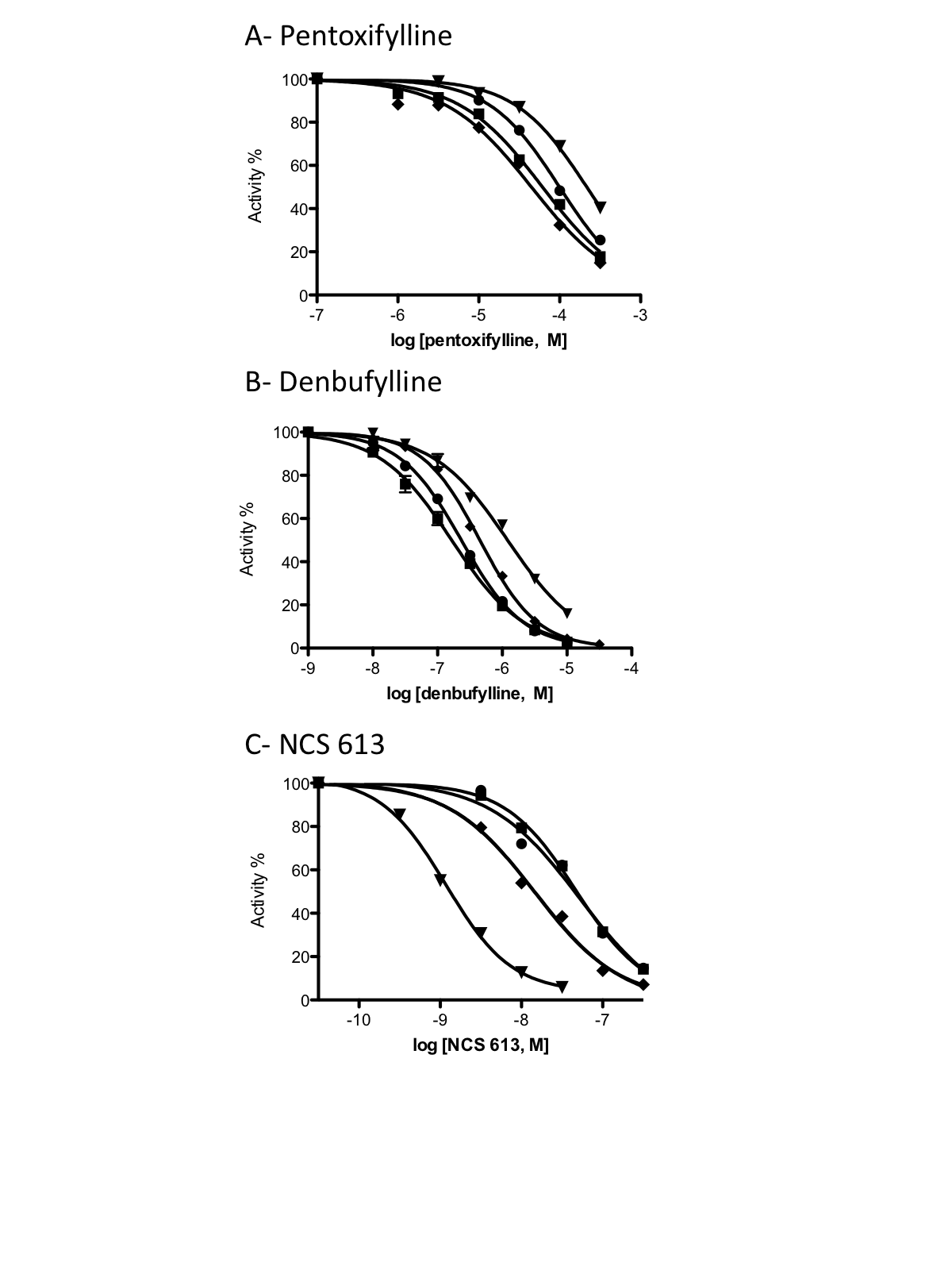

Supplement: Figure S2 — Effects of pentoxifylline (A), denbufylline (B) and NCS 613 (C) on human recombinant PDE4 subtypes: PDE4A (•), PDE4B (▪), PDE4C (▾) and PDE4D (♦). IC50 values were determined as indicated in Materials and Methods . (TIFF) [file pone.0028899.s003.tif]
